# The Queen Square Encephalitis Multidisciplinary Team Meeting - experience over three years, pre and post the COVID-19 pandemic

**DOI:** 10.1016/j.jns.2023.120771

**Published:** 2023-10-15

**Authors:** Tehmina Bharucha, Rachel L. Brown, Cristina Marcoci, Laura Benjamin, Chandrashekar Hoskote, Patricia McNamara, Jennifer Spillane, Michael S. Zandi, Hadi Manji, Queen Square Encephalitis MDT Working Group

**Affiliations:** a National Hospital for Neurology and Neurosurgery, Queen Square, London WC1N 3BG, UK; b Department of Biochemistry, University of Oxford, Oxford OX1 3QU, UK; c University College London, Queen Square Institute of Neurology, London WC1N 3BG, UK; d UCL Institute of Immunity and Transplantation, London NW3 2PP, UK; e UCL Laboratory of Molecular and Cell Biology, London WC1E 6BT, UK; f Lysholm Department of Neuroradiology, National Hospital for Neurology and Neurosurgery, University College London Hospitals NHS Foundation Trust, Queen Square, London WC1N 3BG, UK

**Keywords:** Neurological infections, Encephalitis, Meningitis, Myelitis, Autoimmune disease, Multidisciplinary team

## Abstract

**Background:**

Patients with suspected encephalitis continue to represent a diagnostic and therapeutic challenge, even in highly resourced centres. In February 2018, we set up a monthly in-person multidisciplinary team meeting (MDT). We describe the experience and outcomes of the MDT over three years.

**Methods:**

A retrospective analysis was performed to summarise patient demographics, MDT outcomes and final diagnoses.

**Results:**

Over the three-year period, 324 discussions of 238 patients took place. Cases were diverse; approximately 40% related to COVID-19 or brain infection, 40% autoimmune or other inflammatory disorders and 20% encephalitis mimics or uncertain aetiologies. Feedback from an online survey sent to referring teams and attendees highlighted the value of the MDT; 94% reported the discussion was useful and 69% reported resulting change in patient management.

**Conclusions:**

Multidisciplinary input is crucial in this challenging area, ensuring that all diagnostic avenues are explored and opening doors to novel diagnostics and therapeutics. It also supports clinicians dealing with unwell patients, including in centres where less specialist input is available, and when decisions have to be made where there is little or no evidence base.

## Highlights

• Suspected encephalitis cases are frequently challenging to diagnose and treat, often in evidence-free zones.
• A regular MDT in a tertiary care centre enabled multidisciplinary input with open dialogue and formulation of management plans.
• We urge closer working relationships between centres, and incorporation of referral to these centres in national guidelines for special cases.
• There is also a need for robust collection of observational data from treatment decisions made by the MDT to inform future decision making.

## 1 Introduction

Patients with suspected encephalitis frequently represent a diagnostic and therapeutic challenge, even in the best resourced centres [[1], [2], [3], [4]]. A multitude of potential aetiologies exist, crossing a wide range of disciplines. There is diversity in clinical presentations, and patients are frequently severely unwell requiring intensive care management [5]. Moreover, better patient outcomes are associated with an early diagnosis and treatment, as delays can have devastating consequences [6,7]. Such complex patients need sub-speciality multidisciplinary input, to ensure that known aetiologies are not overlooked, and to ensure there is access to novel diagnostic techniques and therapeutics which may not be widely available [8]. This is apparent in the diagnosis of autoimmune encephalitis, with increasing numbers of new clinically relevant antibodies, and also in the field of infectious encephalitis with, for example, possibilities of metagenomic sequencing to identify potential causative pathogens.

Multidisciplinary team meetings (MDTs) have established roles in streamlining patient care and improving outcomes [9]. The meetings facilitate discussions between multiple specialities, to provide a holistic model of care and develop consensus in the decision-making process. This is most recognised in the field of oncology, but also in a diverse range of other medical and surgical disciplines [9]. In many areas, MDT working is stipulated in guidelines and represents standard care. The meetings also offer educational value. While the structure of meetings are tailored to suit the location and the disease focus, there are suggested good practices for MDTs, such as the incorporation of an experienced chairperson and administrator [9,10]. Furthermore, it is critical that there are processes in place to evaluate the impact of an MDT; negative aspects of MDTs have been described such as time-wastage, attendee fatigue and lack of patient involvement in decision-making, as have issues with poor compliance with MDT outcomes [[11], [12], [13], [14], [15]].

We hypothesised that establishing a multidisciplinary team meeting to discuss complex cases of suspected encephalitis in a tertiary care centre, extended to regional care centres with transfer of patients when indicated, would improve patient outcomes and support clinical teams [1]. In response to this clinical need, in February 2018 we set up a monthly in-person MDT, the ‘Queen Square Encephalitis MDT’, with input from Neurology, Neuroradiology, Infectious Diseases, Microbiology, Virology, Neuroimmunology, Neuropsychiatry, Neuropsychology, Paediatrics, Neuropathology and Neurosurgery [16]. In April 2020, following the onset of the COVID-19 pandemic, with increasing demand and a multitude of novel questions, the MDT was escalated to a weekly virtual meeting open to regional and national referrals. In this manuscript, we aim to describe the process of establishing the MDT, how the meeting impacted on patient management and clinical teams, and the lessons learnt.

## 2 Methods

We performed an observational descriptive analysis of consecutive patients referred to the Queen Square MDT over three years from inception, from February 2018 to January 2021. The National Hospital for Neurology and Neurosurgery is a national tertiary care centre for neurology and neurosurgery for adults in the United Kingdom (U.K.). This included a convenience sample, and no power calculation was performed. A retrospective analysis was performed to summarise patient demographics, MDT outcomes and final diagnoses. This was approved by the Quality and Safety team as a continuous service-evaluation (Reference 140–202,021-SE) registered at University College London Hospitals; for this reason, informed patient consent was not routinely required, but individual patient consent for the expanded cases was obtained. Anonymised feedback was obtained from attendees and referrers using an online survey, detailed in Supplementary Data 1.

The MDT was organised and conducted in a standardised format, described as follows. Referrals were accepted prior to a deadline of 10 am Wednesdays prior to the 1 pm Friday MDT. A proforma, detailing the clinical presentation, investigations, working diagnosis, treatment given to date and MDT question, was completed in advance by the referring team (see Supplementary Data 1). The meeting was chaired by two Consultant Neurologists with expertise in infectious and immune-mediated encephalitis. A representative of the referring team presented the case, with a one slide summary of the case without any identifiable information. There was a 15 min window for the presentation, review of imaging and discussion. Colleagues were asked to raise their hands, or in the virtual meeting to write questions in the ‘chat’. A neuroradiologist demonstrated the imaging findings. Neurology, Infection and Neuroimmunology specialists responded to queries about suspected aetiologies and management in line with current evidence where available [17,18]. Minutes were recorded and outcomes added to the proformas, for example MDT impression and suggestions for additional investigations and/or treatment, which were uploaded to electronic patient records. In April 2020, the structure of the meetings was updated; the monthly in-person meeting was converted to a weekly virtual meeting via Microsoft Teams. A post-MDT meeting is held immediately after the meeting for the core management group to reflect on the meeting and address any issues.

## 3 Results

**Fig. 1:**
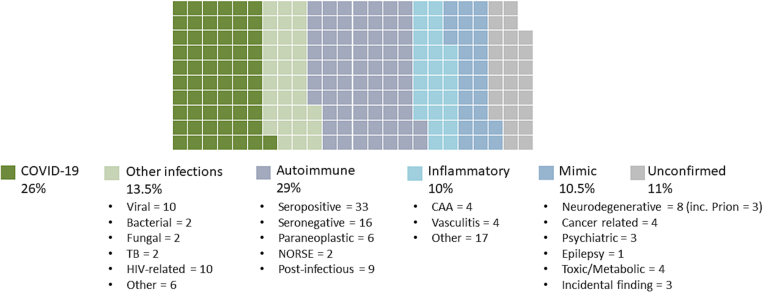
Summary of the diagnoses of included patients discussed at the Queen Square encephalitis MDT.

**Fig. 2:**
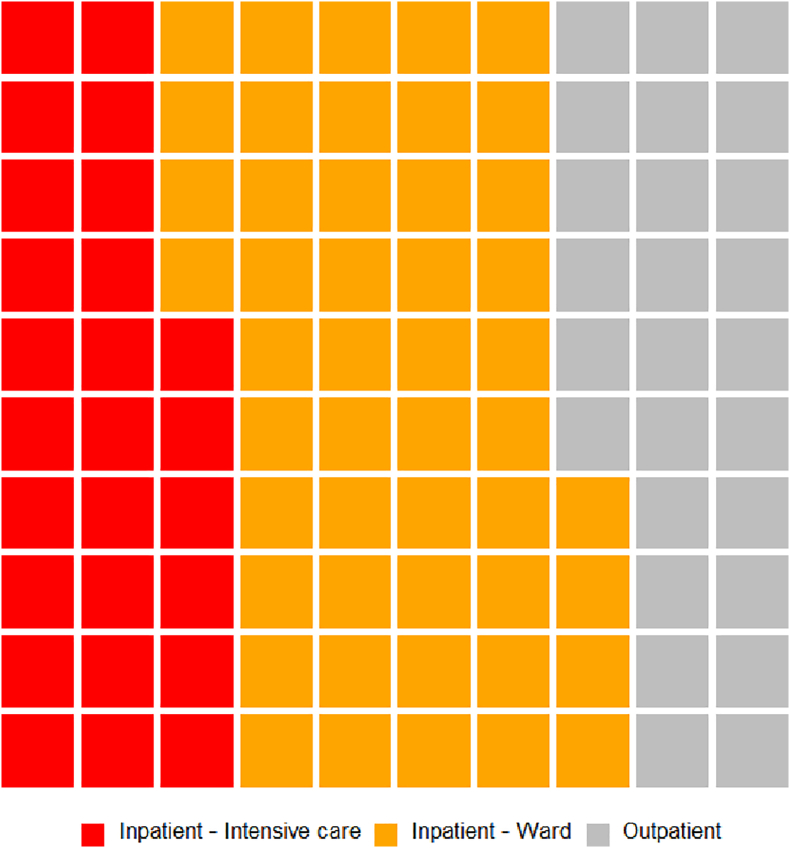
Waffle chart summarising the location of patients discussed at the Queen Square encephalitis MDT.

In the three-year period from February 2018 to February 2021, 324 discussions of 238 patients took place. The median age of patients was 50 years, with a range of 1–91 years, incorporating paediatric referrals when specialist paediatricians from Great Ormond Street Hospital were present. 58% of patients were female. Three-quarters of patients were inpatients and one-third in intensive care, see Fig. 2. Forty-seven percent of referrals were local, managed within University College London Hospitals NHS Foundation Trust (UCLH), 37% were referred from other London hospitals and 16% from hospitals outside London. Questions for the MDT were regarding diagnosis, management or both. Cases were diverse; approximately 40% related to brain infection including COVID-19, 40% autoimmune or other inflammatory disorders and 20% encephalitis mimics or uncertain aetiologies, see Fig. 1. 89% received a confirmed diagnosis. Three cases discussed at the MDT are summarised in Table 1 to illustrate the MDT process, spectrum of cases and potential impact.

**Table 1:** Summary of three illustrative cases discussed at the Queen Square encephalitis MDT.

Case	Background	Question	MDT recommendations	Final diagnosis, management and outcome
1	A 57 year old man had a subacute relapsing meningoencephalitis and persistently abnormal CSF profiles including raised opening pressure, mildly raised protein, normal glucose and white cell counts up to 323, 50% polymorphs. Extensive testing had not revealed underlying infective or inflammatory cause. He was treated for bacterial meningoencephalitis (amoxicillin + ceftriaxone), then later TB therapy and high dose steroids. He improved clinically to near normal with concordant improvement in CSF. He was discharged with ongoing TB treatment and weaning steroid dose but readmitted 4 days later with headache and encephalopathy.	Second opinion on investigation and management	Four MDT discussions over 2 months. Review of clinical history, imaging, brain biopsy histopathology (see Fig. 2, Fig. 3). Recommendations included small bowel biopsy for Whipple's disease, bone marrow biopsy for an underlying lymphoproliferative disorder, CSF metagenomics for untargeted investigation of an infectious aetiology and brain biopsy including for additional viral PCR tests.	Post-infectious cerebral vasculopathy following presumed infective meningoencephalitis (unknown organism). High dose steroids and cyclophosphamide. Very good recovery with return to normal working.
2	A 37 year old woman presented with disinhibited child-like behaviour, dysarthria and dystonic posturing two weeks after uncomplicated SARS-CoV2 infection. Routine blood and CSF studies were normal. Targeted autoimmune screening was awaited at the time of the MDT (later confirmed negative). EEG was consistent with an antibody-associated encephalitis. MRI showed high T2 signal and swelling in the caudate, lentiform nuclei and perirolandic regions.	Advice regarding diagnosis and management	Recommendations included completion of outstanding autoimmune screen and exclusion of toxic and metabolic mimics. Inpatient transfer to specialist centre arranged.	Post-COVID-19 autoimmune encephalitis. High dose steroids with slow taper, and plasmapheresis; quetiapine. Good recovery with minor neurological deficit.
3	A 45 year old woman presented with behaviour change, cognitive decline and ataxia. MRI showed possible abnormal signal in the hippocampi and treatment was initiated for autoimmune encephalitis without benefit. There was a family history of a similar presentation affecting three paternal relatives. CSF RT- QuIC was negative.	Advice regarding diagnosis and management	Two MDT discussions utilising the broad speciality interests and expertise of collaborators within the multidisciplinary team.	Inherited prion disease due to P102l mutation. Multidisciplinary input for the patient and family. Progressive decline in keeping with natural history of disease.

In those with infection, there were a range of viral, bacterial, fungal and parasitic aetiologies, including unusual cases such as tick-borne encephalitis and cerebral malaria. The meetings highlighted the importance of different infection experts thoroughly revisiting the clinical history, for example to consider individual travel exposures and vaccine status. Frequent inputs included recommendation for additional specialised PCR tests such as for astrovirus or geographical PCR panels from the U.K. Health Security Agency's Rare and Imported Pathogens Laboratory at Porton Down. This involved retrieving samples from early in disease presentation or ensuring the correct types of samples were taken when looking for specific infections, for example throat swabs and stool samples for human enteroviruses. Additional testing for intrathecal antibodies sometimes added to the sensitivity of diagnosis where PCR was negative and timepoint was greater than one week from onset of illness - for example in cases of suspected herpes simplex virus encephalitis that were PCR negative. Regular input from the Rare and Imported Pathogens Laboratory, a specialised U.K. centre for the diagnosis of rare, imported or hazardous pathogens, was established. Potentially undiagnosed infections remain a challenge, highlighting the need for ongoing development of diagnostic testing in this area. The importance of CSF and brain metagenomic analysis is recognised, and as a result of MDT discussion we now have access to this novel diagnostic method via collaboration with academic partners [19].

In those with autoimmune disorders, 33 patients had seropositive autoimmune encephalitis (75% of immune-mediated cases), most commonly NMDA-receptor and LGI1 encephalitis (see Fig. 2). MDT discussion in these cases related predominantly to case management. The diagnosis of these encephalitides has greatly improved through increased recognition and rapid turnaround antibody testing. Additionally, these patients were often re-discussed, highlighting the often-challenging long-term management of these patients who may have an incomplete response to first line treatment, prolonged ICU stays and relapses. This MDT format allowed rational and standardised recommendation of additional immune suppression in complex cases where supporting evidence was sparse. Where clinical case management was not covered by available guidelines (rare disorders and complex patients), MDT discussion supported local decision making on therapeutics including treatment with rituximab, bortezomib, tocilizumab, anakinra, use of plasma exchange (PLEX) or immunoadsorption depending upon the diagnosis. The use of pembrolizumab was discussed in four patients with progressive multifocal leukoencephalopathy (PML). Other questions on suspected autoimmune encephalitis related to diagnosis and management of seronegative (16 patients) or paraneoplastic (6 patients) cases, or to the relevance of an incidental antibody without a related syndrome. In the latter case, potentially harmful and unnecessary treatments were avoided.

**Fig. 3:**
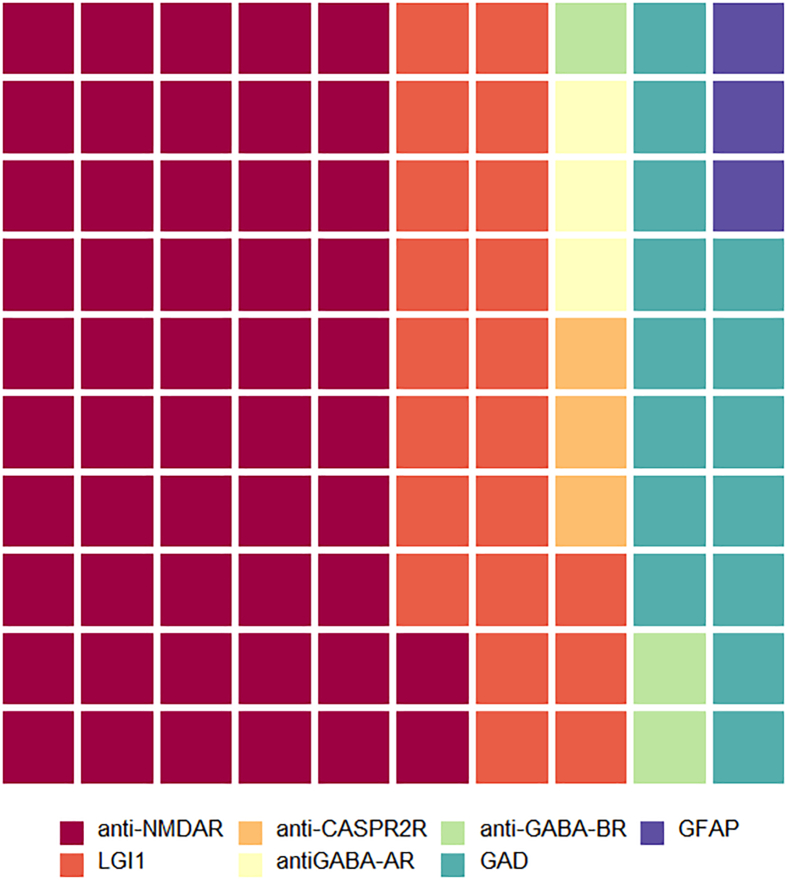
Waffle chart summarising the subcategories of patients with seropositive autoimmune diagnoses discussed at the Queen Square encephalitis MDT.

In the context of COVID-19, several unusual neurological presentations were identified; the MDT became a platform to discuss the many unknowns including patterns of presentations and possible aetiologies. To date, 62 COVID-19 related patients have been discussed. Up to 100 clinicians and clinical scientists attended the meeting per week during the first COVID-19 wave, highlighting the need for clinical guidance and collective agreement on management of patients. In July 2020, we published a case series of COVID-19 related neurological conditions including encephalopathy, encephalitis, ADEM and stroke, resulting from collaborative discussion at the MDT [20]. Detailed neuroradiological input was crucial, particularly when discussing cases involving inflammatory change, unusual strokes and microhaemorrhages (the latter seen frequently). This forum has developed into an important national arena for developing collective expertise in the management of the neurological complications associated with this novel clinical challenge.

Anonymised feedback from an online survey completed by referring teams (*n* = 16) and attendees (*n* = 37) highlighted the value of the MDT; 94% reported the discussion was useful and 69% reported changes in patient management resulting from MDT discussion, detailed in Supplementary Data 2. Themes emerged from the free-text section that participants considered the MDT well-organised, educational and instructive for patient management. We have further developed the meeting as an educational forum, introducing a brief monthly educational update from a specialist team member or invited guest. Topics covered so far have included updates in management of specific infections (HIV, HTLV, PML), stroke, COVID-19-associated disease and the use of diagnostic metagenomics.

## 4 Discussion

We present our experience of setting up an encephalitis MDT in a tertiary care centre in the U.K.. We highlight the complexity of diagnosis and management of these cases, and the need for multidisciplinary input to ensure that all diagnostic avenues are explored and to provide access to novel diagnostics and therapeutic options. The structure of the MDT was comparable to MDTs run in other areas. However, in contrast to other MDTs, there is no guideline specifying that referral to the MDT is standard care, or at what stage it should be made. Overall, the MDT approach supported clinicians managing unwell patients, including centres where less specialist input is available, and when practicing in evidence-free zones.

We saw mimics of autoimmune encephalitis including neurodegenerative diseases such as prion disease, PSEN1-associated young onset Alzheimer's disease. Ayrignac *et al*. have described genetic mimics of CNS inflammatory disease [21] and Flanagan *et al*. have described a high rate of misdiagnosis of cases of autoimmune encephalitis referred to centres [22]. Often this rate reflects specialist services which are biased towards difficult cases without full typical features, or cases which have had no or limited response to conventional therapies. Our own rate of mimics reflects and supports this data and adds some novel disorders.

The prevalence of autoimmune and infectious encephalitis in the era of COVID-19 is difficult to ascertain and requires epidemiological study. We have discussed patients with autoimmune encephalitis shortly after COVID-19, for which there may be a causal relationship, or in which the co-existence of diagnoses may be coincidental, given the high prevalence of COVID-19 at the time of data acquisition.

We acknowledge a number of limitations of the work presented here. While the referrals were received from across the U.K., the MDT is restricted to a single, highly specialised centre, and the findings could not be replicated by many other sites. In line with this, the spectrum of cases is not representative of wider epidemiology on encephalitis, and biased towards unusual, difficult to diagnose and treat cases. There were no clear or systematic guidelines for referral at a national level. The MDT did not record details of the time for discussion of each case that is increasingly acknowledged as an important outcome in MDTs [11]. While psychologists were routinely present, other allied health professionals were not invited such as nurses, physiotherapists, occupational therapists or social workers. Patients were not invited to be present during the MDT, although an encephalitis patient advocat has been present at a meeting and provided feedback on the procedure. We recognise the lack of hard outcomes of death and disability over long-term follow-up, or cost-effectiveness. Systematic follow-up of cases was not performed, not only to review patient outcomes, but also adherence to the MDT decision. We did not explore the preferences of attendees in the move from in-person to remote-working, and acknowledge that there are disadvantages of remote-working such as the potential loss of personal communication and team-working.

The regular MDT has enabled clinicians to present their cases to a multidisciplinary group of experts, enabling open dialogue and formulation of management plans. It is important to emphasise the complexities and heterogeneity of these cases, for which there is rarely an available management protocol. It is for this reason, that these cases need to be discussed among a team, and the expertise requires tertiary care and multi-speciality input. We advocate for wider participation in such meetings, with involvement of international experts where appropriate. There are a few tertiary care centres that could provide similar expertise; we urge development of MDTs and closer working relationships with these centres, and incorporation of referral to these centres in national guidelines for special cases. There is also a need for robust collection of observational data from treatment decisions made by the MDT to inform future decision making.

## Declaration of Competing Interest

MSZ declares honoraria for one lecture each for each of the three mentioned in the last 3 years: Norwegian Neurological Society; Copenhagen Neuropsychological Society, Rigshospitalet; and Cygnet Healthcare. None of the other authors report any conflicts of interest.

## Appendix A Supplementary data

Supplementary material 1: Supplementary Fig. 1: Cortical biopsy with an acute on chronic meningoencephalitis (Case 1 from Table 1) Image 1

Supplementary material 2: Supplementary Data 1: Queen Square encephalitis MDT proforma Supplementary Data 2: Feedback on the Queen Square encephalitis MDT from attenders and attendees. Image 2
