# Supplementary material for: The Queen Square Encephalitis Multidisciplinary Team Meeting - experience over three years, pre and post the COVID-19 pandemic
Source: J Neurol Sci. 2023 Oct 15;453:120771. doi: 10.1016/j.jns.2023.120771 (PMC10951958; doi:10.1016/j.jns.2023.120771)
Supplement: Supplementary file 1 — Supplementary material 1: Supplementary Fig. 1: Cortical biopsy with an acute on chronic meningoencephalitis (Case 1 from Table 1) [file mmc1.docx]

Supplementary Figure 1: Cortical biopsy with an acute on chronic meningoencephalitis (Case 1 from Table 1)

[A] Low power overview with haematoxylin and eosin staining (H&E) - brain tissue comprising full thickness cortex overlying leptomeninges and a small amount of subcortical white matter. [B] H&E - inflammatory cells permeate the vessel wall and in the perivascular space but no fibrinoid necrosis is seen. [C] H&E - in the leptomeninges, there is a mixed diffuse inflammatory infiltrate composed of macrophages, clusters of small lymphocytes, plasma cells, scattered neutrophils and eosinophils. [D] H&E - In the leptomeninges, there is a mixed diffuse inflammatory infiltrate composed of macrophages, clusters of small lymphocytes, plasma cells, scattered neutrophils and eosinophils. [E] Glial fibrillary acidic protein staining - diffuse chronic gliosis. [F] CD68 reveals a moderate diffuse macrophage/ microglia activation with perivascular distribution and cortical microglial clusters.

B

A


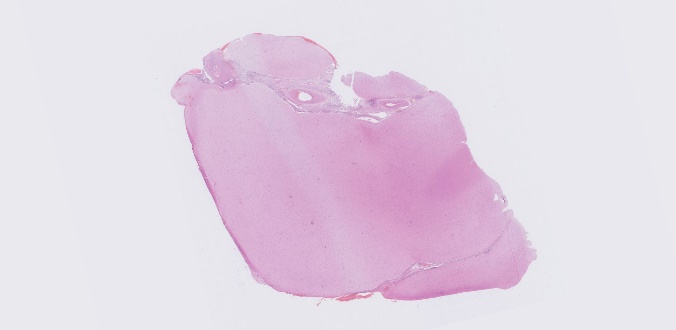

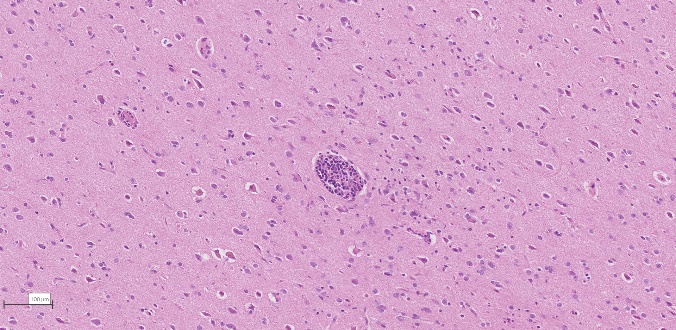


D


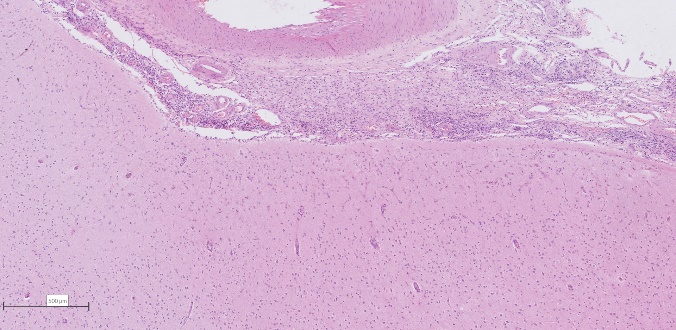

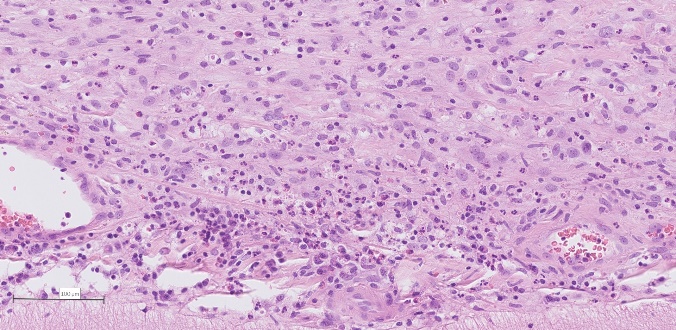


F

C


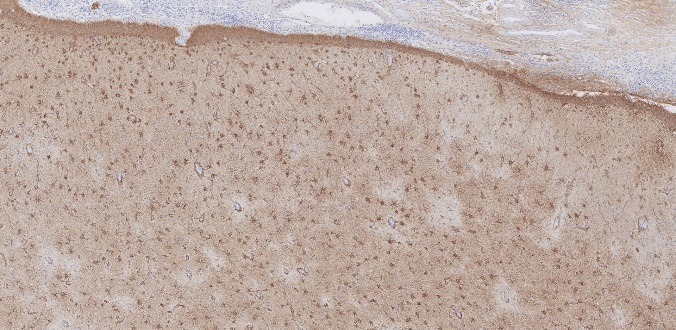

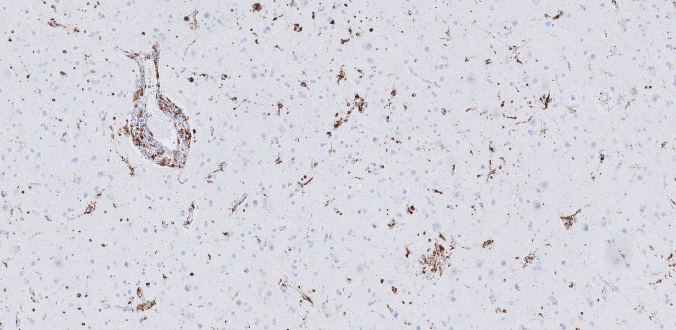


E
