# Supplementary material for: The Queen Square Encephalitis Multidisciplinary Team Meeting - experience over three years, pre and post the COVID-19 pandemic
Source: J Neurol Sci. 2023 Oct 15;453:120771. doi: 10.1016/j.jns.2023.120771 (PMC10951958; doi:10.1016/j.jns.2023.120771)

NHNN Encephalitis MDT – Attendee feedback

37 responses

Accepting responses

Summary Question Individual

How easy did you find the process of accessing the MDT? 0=Difficult to 5=Very easy

37 responses

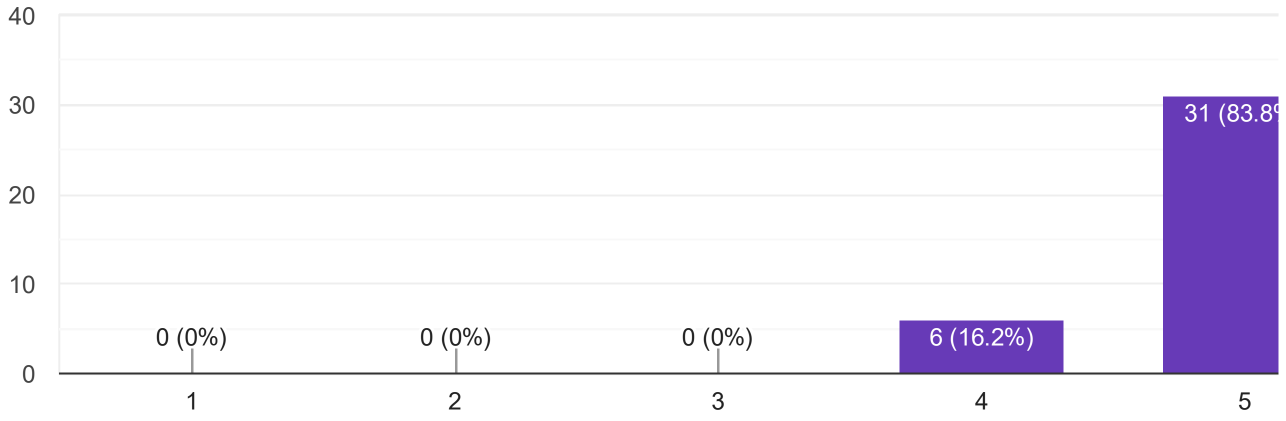

[illegible]v educational did you find the discussion? 0=Not educational to 5=Highly educational

37 responses

Questions Responses 37 Settings

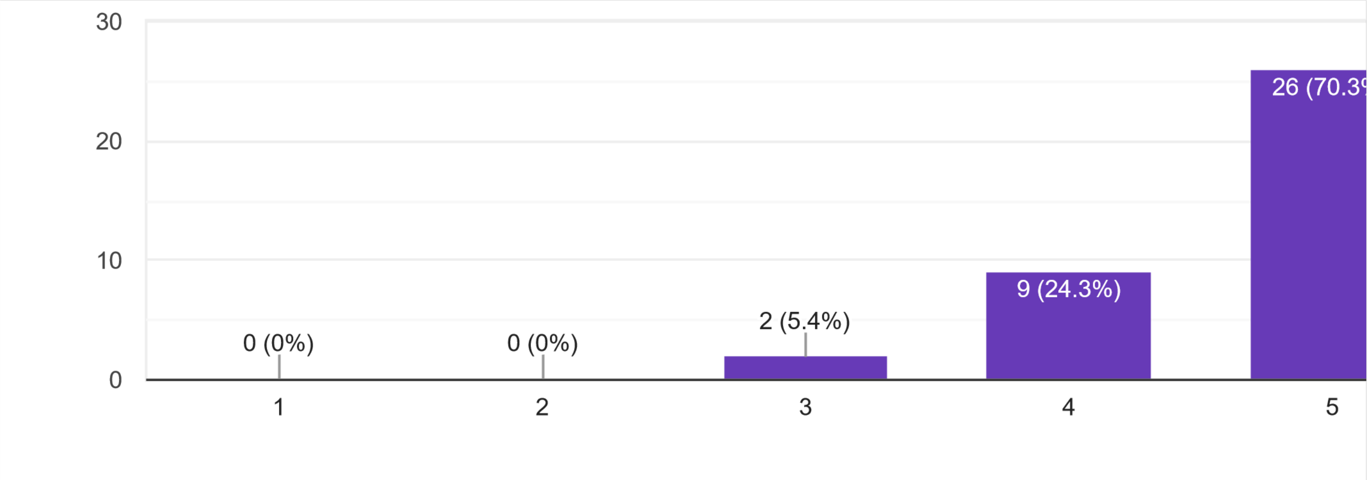

How useful did you find the monthly 15 minute educational update seminars? 0 = Not useful to 5 = Very useful

28 responses

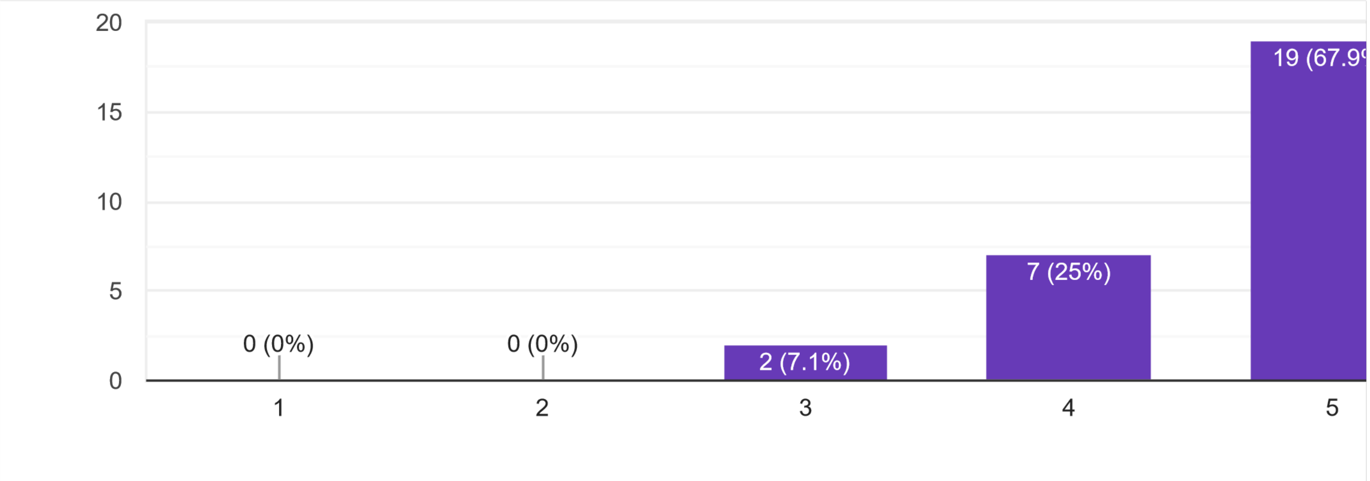

v regularly do you attend?

37 responses

Questions Responses 37 Se ings

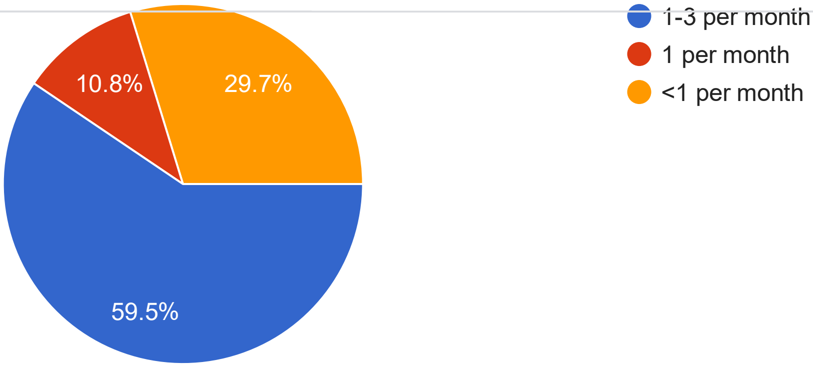

NHNN Encephalitis MDT - Referrer's Feedback

16 responses

Publish analytics

How easy did you find the process of referring a patient? 0=Difficult to 5=Very easy

16 responses

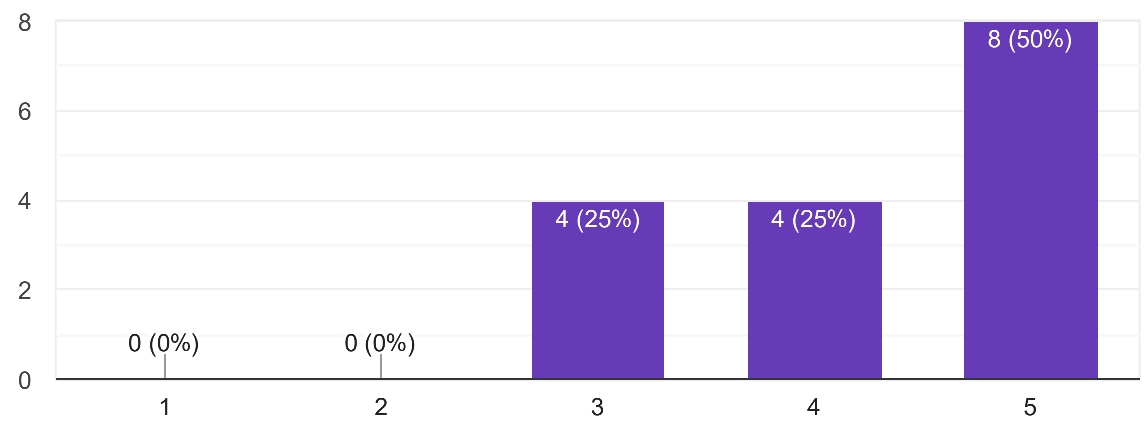

How useful did you find the discussion? 0 = Not useful to 5 = Very useful

16 responses

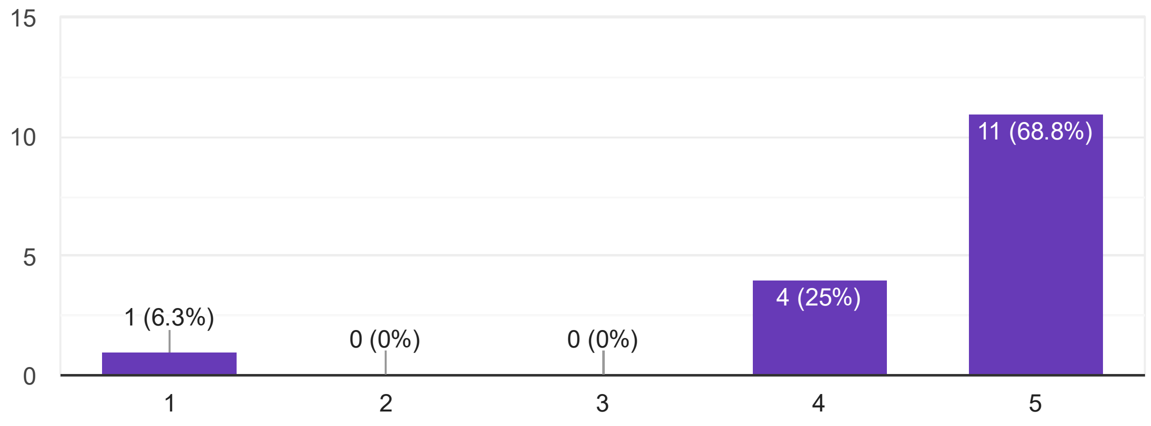

Did it change the management of your patient

16 responses

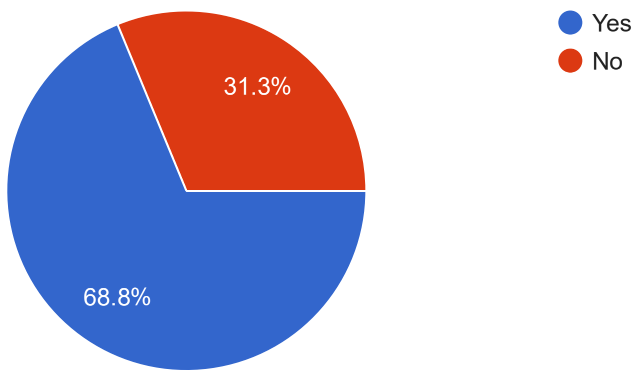

Would you refer another patient?

16 responses

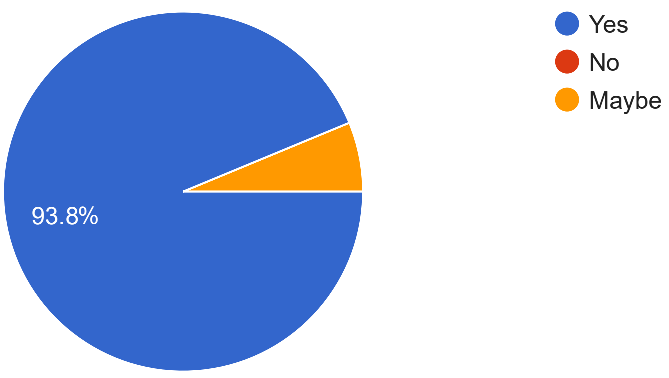

Supplement: Supplementary file 2 — Supplementary material 2: Supplementary Data 1: Queen Square encephalitis MDT proforma Supplementary Data 2: Feedback on the Queen Square encephalitis MDT from attenders and attendees. [file mmc2.pdf]
